# An Investigation of Dairy Cattle Welfare in Commercial Iranian Farms: Results from Animal- and Stockperson-Based Measures †

**DOI:** 10.3390/ani15030359

**Published:** 2025-01-27

**Authors:** Ali Jafari-Gh., Richard Laven, Fatima Khaloubagheri, Mohsen Haji Mirrahimi, Saeid Jafari-Gh., Mehdi Dehghan Banadaky, Kristina Ruth Mueller, Emilie Vallee

**Affiliations:** 1 School of Veterinary Science, Massey University, 4410 Palmerston North, New Zealand; 2 Independent Researcher, 4472 Palmerston North, New Zealand; 3 School of Veterinary Medicine, Karaj Islamic Azad University, Karaj 3149968111, Iran; 4 Animal Science Department, College of Agriculture and Natural Resources, Karaj Islamic Azad University, Karaj 3149968111, Iran; 5 Animal Science Department, College of Agriculture and Natural Resources, University of Tehran, Karaj 3158777871, Iran

**Keywords:** animal welfare, cow comfort, ear damage, tail damage, lameness, hock lesions

## Abstract

Dairy cow welfare can impact both the economic performance of an individual farm and the public perception of the sector, thus influencing the sustainability of the dairy farming industry. Intensive dairy farming in Iran started in the 1940s, but there is limited data available regarding dairy cattle welfare in the country. Therefore, the aim of this study was to design and use a comprehensive bespoke welfare assessment protocol in intensive Iranian dairy farms to provide baseline data for Iran and other countries with similar farming systems. The protocol consists of animal-based, stockperson-based, and resource-based measures. The first part of the welfare assessment protocol (being presented in this paper) was developed from the existing literature and contained 11 animal-based and 2 stockmanship measures. The protocol was applied in 62 intensive dairy cattle farms in five arid and semi-arid provinces of Iran. Welfare Quality sample size suggestions were used to reach the minimum sample size for all animal-based measures except for the locomotion score, where either the whole milking herd was scored or all cows in the early lactation (<150 days in milk) group. Results show that there was a good focus on nutrition management on the farms. Lameness prevalence and the incidence of hock lesions were too high, although within the range seen in many housed cows outside Iran. In addition, 13.5% of cows had damaged ears. The prevalence of dirty legs was high (≥80% of cows), and the hygiene score was unrelated to the farming system. Finally, the extremely high median prevalence of tail damage (60%) indicates a significant welfare issue that needs to be addressed with urgency.

## Simple Summary

Domestic cattle have been a significant part of Iranian agriculture since at least the early-to-mid sixth millennium BCE. Farmers traditionally follow some animal welfare practices, which mainly focus on treating animals with respect and avoiding practices such as tail docking or tethering. However, intensive dairy farming has been present for a shorter period of time, yet there have been no systematic assessments of animal welfare in this non-traditional farming industry. Exploiting current welfare protocols and studies in Europe, New Zealand, and North America, this project aimed to design a welfare assessment protocol that can be suitable for Iranian intensive dairy farming and used it to assess cattle welfare on 62 farms. Although a one-time welfare assessment is not reflective of the actual welfare of the cows, this study aimed to create baseline data for Iran and other countries with similar farming systems. These results show some areas of welfare, such as nutrition management, are managed well on Iranian dairy farms, but there are areas that need more attention, such as lameness management, hock health, and tail damage. Overall, our results indicate that Iranian dairy farmers would hugely benefit from more education regarding dairy cattle welfare.

## 1. Introduction

Dairy cattle welfare is crucial to the sustainability of the dairy farming industry as it can influence both the economic performance of an individual farm [1] and the public perception of the sector [2,3,4]. Indeed, animal welfare is of paramount importance to sustain the social license of dairy farming, as consumers and other stakeholders expect dairy cattle to have, at least, a life worth living [2,4].

Independent welfare assessment is used as a means of demonstrating that on-farm welfare is meeting or exceeding standards by using animal-based, resource-based, and stockperson-based measures to assess, either directly or indirectly, the welfare of the animal [5]. Therefore, farm assurance schemes (e.g., AssureWel and Red Tractor) are now widely used in many, though far from all, countries [6,7,8,9].

Domestic cattle have been a significant part of Iranian agriculture since at least the early-to-mid sixth millennium BCE [10]. As such, there is a considerable amount of ‘traditional practice’ related to cattle rearing in Iran. In relation to animal welfare, this practice is focused on treating animals with respect and avoiding practices such as tail docking or tethering [11,12]. On the other hand, intensive dairy farming (i.e., a system in which animals are kept in a confined environment, consume high amounts of grains, and are fed principally on preserved forages rather than grazed grass) has been present for a much shorter period of time (~80 years) [13]. To date, there have been no systematic assessments of animal welfare in this non-traditional farming industry, even though the Iranian government is focused on increasing the export of dairy products [13], and recent research has shown that Iranian consumers are demanding and willing to pay for high dairy cattle welfare standards [14].

Therefore, the aim of this study was to develop a comprehensive welfare assessment protocol that included animal-based, resource-based, and stockperson-based measures, alongside farm records, on intensive Iranian dairy farms and to use this protocol to systematically assess welfare on Iranian dairy farms and produce baseline welfare data for dairy cattle in Iran. This paper presents the development of that protocol and a descriptive report of the outcomes of the assessment of animal- and stockperson-based welfare measures.

## 2. Material and Methods

The Animal Ethics Committee of the College of Agricultural and Natural Resources, University of Tehran, approved all procedures used in this research.

### 2.1. Development of the Welfare Assessment Protocol

Since intensive dairy farming in Iran is very close to the North American system, multiple papers published in that region [15,16,17,18,19,20], plus some European [21,22,23] and NZ studies [9,24,25], were used as baseline data for the development of the protocol. In addition, other European welfare protocols, such as the Welfare Quality [26] and AssureWel [27] assessments, were consulted. Measures that could potentially be assessed on intensive Iranian dairy farms were listed by the research team, who then created a shortlist of the measures that they thought would be essential to assess. This shortlist was then used to create an assessment tool that was tested and finalised, alongside the calibration of the assessors using the University of Tehran dairy cattle farm (Karaj, Iran). Additional assessments could be added to the protocol during the visits if the research team identified an area of interest that was not covered by the protocol. Thus, not all the assessments were undertaken on all 62 farms.

### 2.2. Testing and Finalisation of Welfare Assessment Protocol and Calibration of Assessors

Testing of the feasibility and practicability of the assessment process took three months (February to April 2022). It was undertaken alongside the calibration of the research team (i.e., comparing scores and discussing differences). Where thought necessary (locomotion scoring and tail scoring), the scoring process was filmed, and the film and the individual scores recorded by the team members were sent to R.L. (a researcher who has significant experience in the scoring systems being used). He provided commentary on the scoring to ensure that the research team reached a common understanding about the scoring systems (e.g., how a locomotion score 2 cow moves different parts of her body, or how different types of tail injury present). The dataset from this process was not included in any analysis; however, the university farm was formally assessed later.

### 2.3. Study Population

The aim was to visit at least 60 intensive dairy farms [23] from five key dairying provinces in Iran: Tehran, Isfahan, Alborz, Qom, and Khorasan. A convenience selection was made with snowball sampling used to increase the probability of a selected farmer agreeing to be included [28]. Before any data collection, it was explained to the farmer that any data gathered would be anonymised and only published in an aggregated report. Verbal consent was obtained before the assessment started.

### 2.4. Sample Size

The whole lactating herd was initially chosen as the target population. For locomotion scoring only, all lactating cows were scored, and for other assessments, the recommendations by Cook [29] were used to identify a sample size that would identify the true prevalence with 95% confidence to a precision of 5% (assuming true prevalence of 50%). After five farm visits, it was concluded that this sample size was too large, limiting the number of farms that could be assessed within the timeframe available and limiting the value of the study as a model for future welfare audits. Thus, the target population was changed from all lactating cows to cows in early lactation (days in milk [DIM] < 150). The exception to this was on farms where cows were not grouped based on DIM. Again, for locomotion score, the whole target population was scored, and for other assessments, sample size was calculated in order to identify true prevalence with 95% confidence to a precision of 15% (expected true prevalence of 50%; [26]).

### 2.5. Farm Visits

Farm visits started in May 2022 and finished in March 2023. The aim was to visit each farm only once and for data gathering to take one working day. The visits started by asking a set of questions about the farm, such as the total number of animals, the number of the lactating herd, their management routines, and by asking the farmer to show the research team different sections of the farm, e.g., the calving area, milking shed, and group pens. Relevant comments made by the farmer during the process were recorded informally before the farm assessment.

### 2.6. Data Gathering Process

The data gathering and handling team consisted of four members: A.J-Gh. (animal scientist), F.K. (farmer, agronomist), S.J-Gh. (animal scientist), and M.H.M. (senior veterinary medicine student). A.J-Gh was the sole person responsible for locomotion scoring, body condition scoring, hock lesions, and tail scoring. Other measures were scored by the other team members based on their availability and workload.

### 2.7. Animal-Based Measures

**Figure 1:**
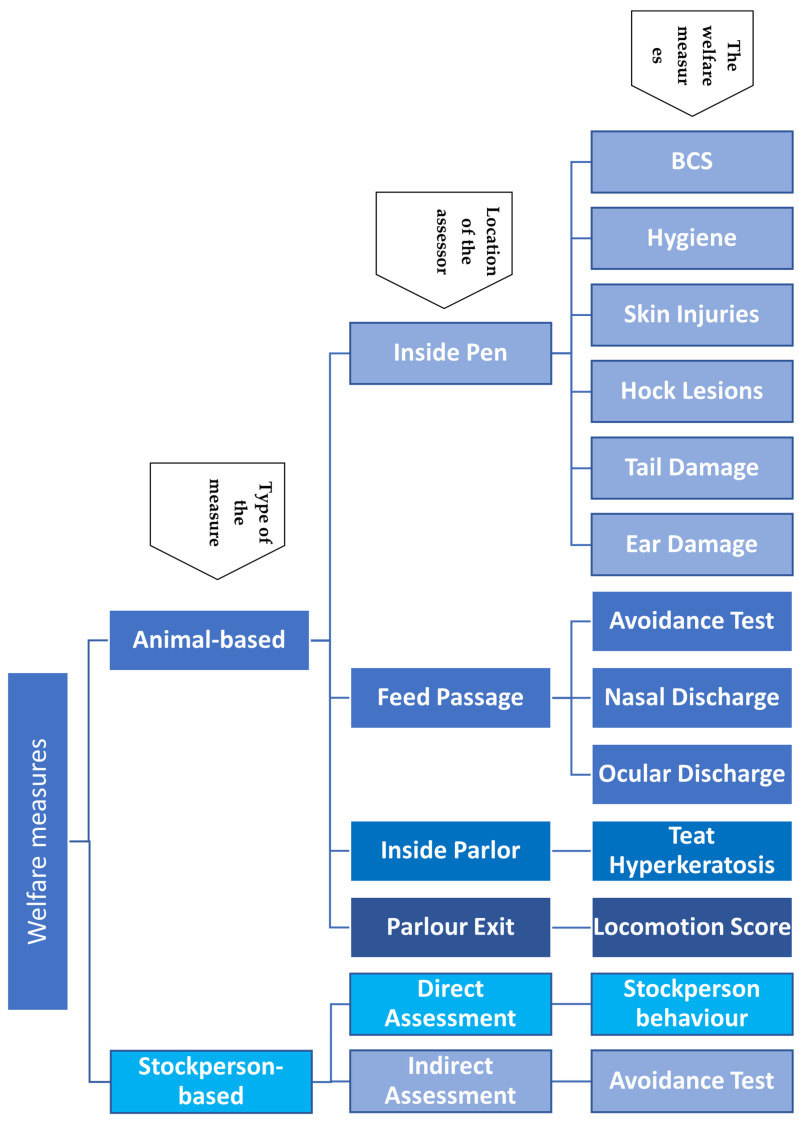
Animal- and stockperson-based measures assessed and the location of the assessments.

Measures were divided into different groups based on the part of the farm where the assessment was completed (see Figure 1). For the measures assessed in pens, scoring was undertaken around feeding time when most cows were standing.

Only cows standing in the feed alley (free stall farms) or the walking area (bedded-pack farms) were sampled. Cows that were lying down were excluded to minimise disturbance. If the number of eligible cows exceeded the sample size needed (i.e., N), animals were sampled systematically. For free stall farms with a feed alley, the alley was mentally divided into three sections, and N/3 cows were sampled in each of those sections. For bedded-pack farms, the walking area was divided into four sections, and N/4 cows were sampled per section. For each section, the assessment started with the animal closest to the start of that section, and every other animal was scored until N/3 or N/4 animals had been scored (or the next whole number). For these in-pen assessments, cows that were clearly nervous when approached (i.e., had a large flight zone) were excluded from assessment.

### 2.8. Scoring Systems

#### 2.8.1. Measures Assessed with the Assessor in the Pen

These were BCS, body hygiene, tail damage, skin lesions, ear damage (the latter was assessed from the fifth farm onwards only), and hock health (from the eighth farm onwards only). BCS was scored visually using the 1 to 5 UK system [30] with a score < 2 identifying a cow that was too lean and a score > 4 a cow that was too obese.

Body hygiene was scored separately in the lower leg, upper leg, udder, and tail using a three-point (0 to 2) system [31]. A cow was considered too dirty if the score was 2 (i.e., ≥25% of the area was dirty).

**Figure 2:**
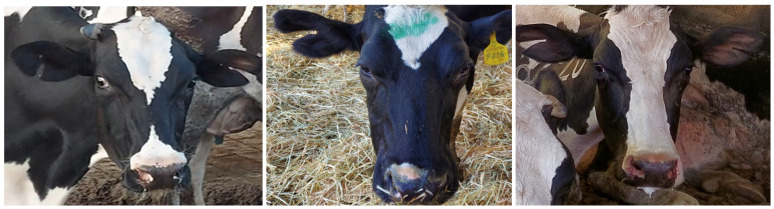
Examples of cows with ripped ears. This was considered ear damage in this study.

Both sides of the body were observed and scored (0–3) for skin injuries as per Cook [29], with a score ≥ 2 indicating significant damage. Ear damage was recorded based on visible signs of any tears in the ears as a presence/absence (Figure 2).

Hock (dorsal joints) lesions were assessed by separately recording signs of hair loss, ulceration, and swelling on a 4-point scale [32]. Any score of ≥2 was recorded as significant hock damage.

Tail damage was visually assessed using a modified version of the New Zealand Veterinary Association tail scoring system [33]. Briefly, the tail was divided into thirds (three equal zones) from its top (zone 1) to the bottom (zone 3). Three forms of tail damage were recorded: trauma (T), breakage (B), and shortened tail (S). Swelling of the tail, not associated with skin damage, was recorded as ‘breakage’ alongside tail deviation. All lesions and the zone affected were recorded.

#### 2.8.2. Measures Assessed with the Assessor in the Feed Passage

These included ocular and nasal discharge and avoidance tests (from the 10th farm onwards). Discharges were recorded based on the Wisconsin-Madison three-point system, with a score of 2 being considered as significant discharge [34]. An avoidance test was performed as per Crossley et al. [35]. Briefly, the assessor slowly (one step per second) approached the cows standing at the feed face. Scores of 2, 1, and 0 were given if the cow moved away with the assessor being ~>1 metre away, if the assessor was closer than 1 metre but before extending the hand toward the cow, and if the cow allowed the assessor to extend the hand or touch her, respectively.

#### 2.8.3. Measures Assessed with the Assessor in the Milking Shed

Teat hyperkeratosis was assessed from the 13th farm onwards using a 1–3 scoring system [36]. All four teats were scored, and the highest individual score was assigned to the cow. Cows with a score ≥ 2 were recorded as having significant hyperkeratosis.

#### 2.8.4. Measures Assessed with the Assessor in the Parlour Exit

Locomotion scoring was performed at the parlour exit on cows returning to their pens after milking using a 4-point scale [37]. Cows were recorded as lame if the score was ≥2 and severely lame if the score was 3.

### 2.9. Stockperson Behaviour

Stockperson behaviour was assessed visually during milking time and when the cows were taken from the pen to the collecting yard. The researcher stayed in a nearby pen to keep a reasonable distance to avoid interfering with stockperson routines to ensure they behaved as naturally as possible. The stockperson was not informed that their handling skills were being monitored. Stockperson behaviour was classified as 0 (not using force to move cows), 1 (moving cows using loud noises or hitting with tools), or 2 (moving cows with both loud noises and hitting).

### 2.10. Data Handling and Statistical Analysis

All data were recorded on bespoke paper forms (see Supplementary Files) before being transferred to Microsoft Excel spreadsheets. Data were checked to ensure that no transfer errors or misrecordings were made. SAS version 9.4 (SAS Institute, Cary, NC, USA) was used for all analyses except where otherwise stated. Descriptive statistics (mean, 95% confidence intervals for mean, minimum, maximum, median, and interquartile ranges) were calculated for all measures, and boxplots were created for measures divided by farm system (free stall vs. bedded pack) and farm size (categorised using tertiles). For illustration purposes, where it was thought to be useful to further evaluate the effect of farming system or farm size on a welfare outcome, a generalised linear mixed model (binomial distribution with a logit link) was used with prevalence of welfare as the outcome and farm size or system as the predictor variable and farm as a random effect. This modelling was undertaken using SPSS version 29 (IBM, Armonk, NY, USA).

## 3. Results

### 3.1. Overview of the Visits

Overall, 94 farmers were contacted, and 63 farms were visited in the five provinces: Tehran (n = 37), Alborz (n = 15), Isfahan (n = 3), Qazvin (n = 4), and Qom (n = 4). Between May and September 2022, 58 farmers were approached, and 53 agreed to be visited. After the death of Mahsa Jina Amini on 16 September 2022, nationwide pro-human rights uprisings followed, and the study paused for 4 months. Farmers were contacted again from January 2023, with 36 being approached but only 10 agreeing to a visit.

Separating farms into three size categories using tertiles resulted in small farms having ≤180 lactating cows, medium farms having 181 to 899 lactating cows, and large farms having ≥900 lactating cows. Data related to one small farm were lost, so data were available for analysis from 62 farms.

On the first five farms, data collection (sample size as per Cook [29], with four assessors), took between 2–4 working days per farm. After reduction in sample size to that recommended by Welfare Quality [26], all assessments were completed in one working day with two assessors. Table 1 shows the number of animals scored for each measure and the number of farms assessed. Table 2 shows the distribution of the farm-level prevalence of animals with different welfare issues across all assessed farms. 

### 3.2. Nutrition

The median (interquartile range; IQR) herd-level BCS was 2.75 (2.75 to 2.88). Maximum herd-level prevalence of cows with a BCS of <2 and >4 was 7.8% and 5.1%, respectively (Table 2).

### 3.3. Physical Environment

#### Body Hygiene

**Figure 3:**
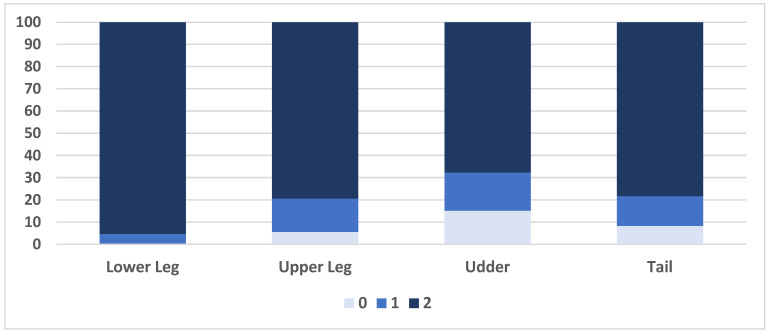
Relative frequency of each hygiene score in different parts of the body in a cross-sectional study of 62 Iranian intensive dairy farms. Score 0: <5% of the body part is dirty; score 1: 5 to 25% of the body part is dirty; score 2: >25% of the body part is dirty.

The lower part of the leg was the dirtiest part of the body (median farm level prevalence: 100%), while the udder was the least dirty part (median farm level prevalence: 83%; Table 2; Figure 3).

Prevalence of dirty body parts was apparently unrelated to the farming system. Farm-level median (IQR) prevalence of dirty body parts in free-stall (FS) vs. bedded-pack (BP) farms were 100% (99.5% to 100%) vs. 100% (98.6% to 100%) for lower legs, 91.2% (72.4% to 97.1%) vs. 97.1% (86.7% to 100%) for upper legs, 71.8% (44.8% to 88.7%) vs. 90.0% (66.7% to 98.6%) for udders, and 95.4% (79.4% to 98.8%) vs. 94.6% (80.0% to 98.6%) for tails, respectively.

### 3.4. Health

#### 3.4.1. Tail Damage

**Figure 4:**
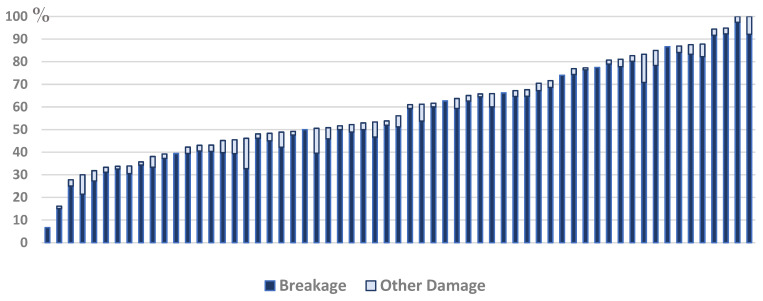
Prevalence of damaged tails in 62 Iranian dairy farms in a cross-sectional welfare assessment study. Breakage includes swellings and deviations; other damage includes trauma and shortened tails.

All farms had cows with damaged tails. Farm-level prevalence ranged from 6.7% to 100% (Figure 4; Table 2).

**Figure 5:**
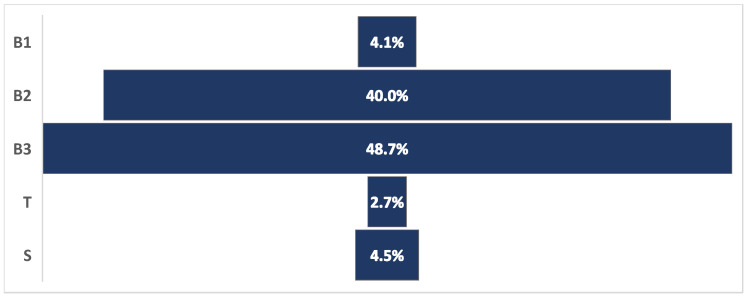
Relative frequency of different types of tail damage in a cross-sectional welfare assessment study of 62 Iranian dairy farms. B1: breakage in zone 1 of the tail (i.e., the top one-third of the tail); B2: breakage in zone 2 of the tail (i.e., the middle one-third of the tail); B3: breakage in zone 3 of the tail (i.e., the lower one-third of the tail); T: trauma; S: shortened.

Breakage was the most prevalent form of tail damage, accounting for 92.8% of all tail problems, with 4.5% of animals with tail damage having shortened tails and 2.7% having trauma (Figure 5).

**Figure 6:**
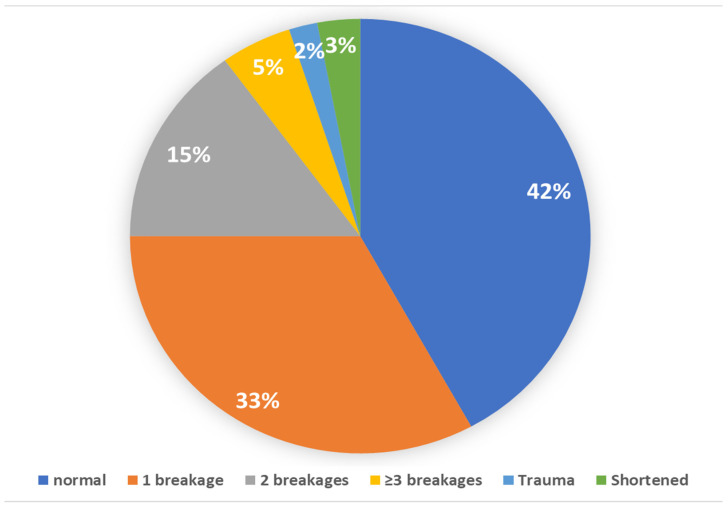
Proportion (cow-level prevalence) of animals with different tail conditions in a cross-sectional welfare assessment study of 62 Iranian dairy farms.

Of the 4796 cows who were scored for tail damage, 33% had one breakage, 15% had 2 breakages, and 5% had ≥3 (see Figure 6 for cow-level prevalence of tail damage). Our data were compatible with no effect of farm size or farming system on the prevalence of tail damage (see Table 3 and Table 4).

#### 3.4.2. Locomotion Score

**Figure 7:**
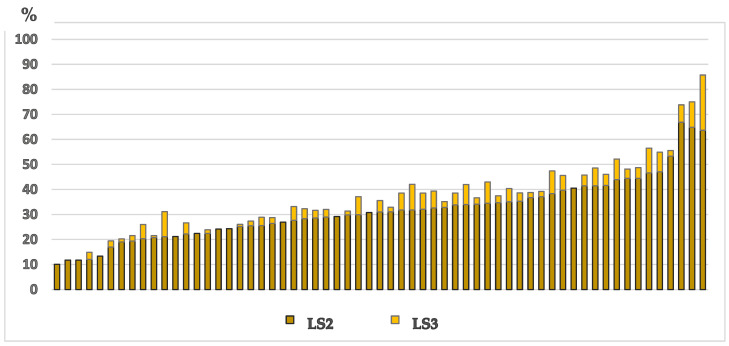
Prevalence of lameness in 62 Iranian dairy farms in a cross-sectional welfare assessment study. LS2: locomotion score 2 out of 3 (i.e., clinically lame cows); LS3: cows with locomotion score 3 out of 3 (i.e., severely lame cows).

The median (IQR) within-farm prevalence of lameness (cows with locomotion scores 2 and 3; ≥LS2) was 32.9% (26.0% to 42.1%). Table 2 shows the prevalence of LS2 and LS3 cows. There were no severely lame animals (LS3) on 12/62 farms (Figure 7). Of these 12 farms, ten had ≤180 lactating cows, and two had ≥900 lactating cows. Nevertheless, our data were consistent with no effect of farm size or farming system on the prevalence of lameness (Table 3 and Table 4).

#### 3.4.3. Hock Lesions

**Figure 8:**
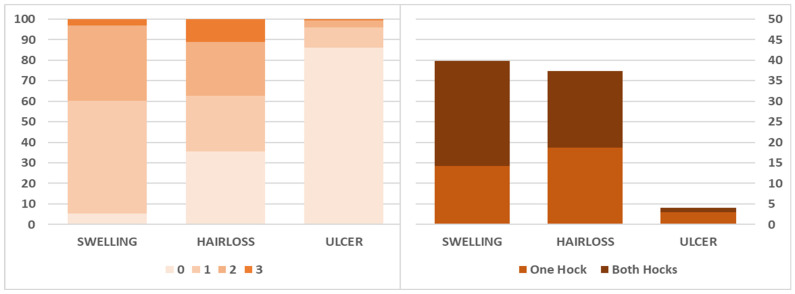
Relative frequency of hock lesion scores (**Left**) and farm prevalence of severe hock lesions in either the left or right foot only or in both feet (**Right**) in a cross-sectional welfare assessment of 62 Iranian dairy farms. Scoring was completed as per Potterton et al. [32], where score 0 means an absence of a lesion on the hock (dorsal joint), score 1 is mild lesions, and scores 2 and 3 show severe lesions.

Overall, median (IQR) prevalences of hair loss on the left hock alone, right hock alone, or both hocks (Table 2; Figure 8) were 8.1% (5.9% to 11.8%), 3.9% (2.8% to 7.3%), and 17.5% (6.6% to 35.0%), respectively. The same figures for swelling were 7.5% (4.4% to 13.3%), 1.7% (0.0% to 3.2%), and 24.1% (13.3% to 45.5%), respectively, and for ulcer were 0% (0% to 1.9%), 0% (0.0% to 1.7%), and 0% (0% to 0.9%), respectively.

**Figure 9:**
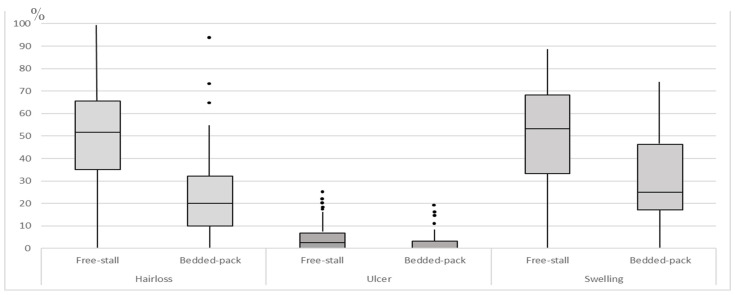
Distribution of prevalence of hock lesions in free-stall and bedded-pack farms in a cross-sectional study of 62 Iranian dairy farms.

The effect of farm system on hock lesions is summarised in Figure 9. Compared to BP farms, odds of having hair loss, ulcers, and swollen hocks on FS farms were all higher (OR 3.82 (95% CI: 2.37 to 6.13), 2.35 (95% CI: 1.18 to 4.70), and 2.56 (95% CI: 1.60 to 4.10), respectively). In contrast, our data were consistent with no effect of farm size on the prevalence of any hock lesion type (Table 3 and Table 4).

#### 3.4.4. Injuries on Knees, Back, Neck, and Other Body Parts

Table 2 summarises the prevalence of integument alterations and ear damage across the farms. Back and neck injuries had the lowest prevalence (median farm-level prevalence of 3.5% and 4.3%, respectively).

#### 3.4.5. Ear Damage

The median (IQR) of ear damage across all farms was 13.5% (7.3% to 25%). While there were 7 farms with <5% prevalence of damaged ears, 3 farms had a prevalence of >50%.

#### 3.4.6. Nasal and Ocular Discharges

The median (IQR) within-farm prevalence of cows with significant nasal and ocular discharges was 6.2% (1.7% to 11.6%) and 12.8% (6.5% to 19.7%), respectively (Table 2). There were 10/58 and 3/60 farms that had no cows with significant nasal or ocular discharges, respectively.

#### 3.4.7. Teat Hyperkeratosis

The median (IQR) within-farm prevalence of cows with severe teat hyperkeratosis was 8.7% (4.2% to 42.3%; Table 2). There was a wide variation in farm-level prevalence of teat hyperkeratosis (ranging from 0% to 91%) with only one farm having no cows with hyperkeratotic teats.

#### 3.4.8. Stockperson Behaviour (Direct Measurement of Human-Animal Relationship)

Direct measurement could not be completed on 2/62 farms as the time of measurement coincided with other welfare assessments. In 8/60 farms handling with no hitting or loud noise was observed, while 15/60 farms used either hitting or making loud noises. In 37/60 farms, stockpersons used both hitting and shouting/loud noises, and in 3/60 farms, violent behaviour was observed (i.e., hitting the animal in sensitive parts such as the head, or hitting too hard to deliberately hurt the animal).

#### 3.4.9. Avoidance Test (Indirect Measurement of Human-Animal Relationship)

Table 2 shows the within-farm prevalence of animals with different human-animal interactions. The median (IQR) within-farm prevalence of cows that allowed the researcher to touch them (avoidance 0) and those who had high flight zones (avoidance 2) were 38.9% (28.0% to 53.0%) and 36.4% (21.1% to 46.6%), respectively.

## 4. Discussion

This is the first systematic assessment of welfare on Iranian dairy farms. Previous studies have assessed some of the animal-based measures assessed in this study, such as BCS [38,39] and lameness (e.g., [40]), but no previous study has recorded and compared multiple welfare measures on individual farms. This lack of a focus on welfare assessment was reflected in the lack of knowledge of the term ‘animal welfare’ by many of the farmers involved in this study, with the term “welfare” (In Persian: Refa`h; رفاه) often being confused with “comfort” (In Persian: Asayesh; آسایش). This familiarity with the term ‘comfort’ rather than animal welfare may be related to the regular “Cow Comfort and Lameness” congress that has been held regularly in Iran since 2015.

### 4.1. Nutrition

In this dataset there were very few cows that were too lean or too fat. This is unsurprising, as for intensively managed dairy cattle, ensuring feed availability is an essential part of optimising milk production [41], and 61/62 farms employed a full-time nutritionist or a nutrition consultant. Furthermore, measurement of BCS has long been used as a practical management tool on Iranian dairy farms, with target BCS of 3.5 to 4 in fresh cows and 3 to 4 in other production groups (markedly different from our welfare thresholds).

Body condition score was the only directly nutrition-related, animal-based measure included in the assessment. Since BCS is reflective of medium/long-term nutrition status of the animal, it is often recommended to be used alongside shorter-term indicators, such as rumen fill score [42]. However, much of the variation in rumen fill score is not related to feed intake, limiting its value as a welfare measure.

### 4.2. Physical Environment

#### Body Hygiene

This study reported a high prevalence of dirty animals consistent with (although at the high end of) results from housed cattle across the world [43,44,45]. Despite free stall systems being designed to improve cow cleanliness [46], we found no clear difference at the univariable level between farm systems in the proportion of dirty cows. This suggests in both systems that walking alley cleanliness on many Iranian dairy farms is inadequate and that existing cleaning technologies are often ineffective. Our data strongly suggest that more attention needs to be paid to farm hygiene on many Iranian dairy farms.

### 4.3. Health

#### 4.3.1. Tail Damage

Few studies have reported the prevalence of tail damage in dairy cows, with the reported prevalence of tail damage varying from 4% on Uruguayan dairy farms [47] to 46% in a study completed on a single US dairy farm [48]. Although direct comparison across studies is complicated by the lack of precise definitions of tail damage, the use of observation in some studies and palpation in others, and the inclusion/exclusion of docked tails. The median herd-level prevalence of damaged tails in our study population was 60%. This is much higher than any other previous report. In particular, it is much higher than the 14.9% reported by Cuttance et al. [49], who used a modified NZVA tail score. This difference may actually be larger as Cuttance et al. [49] used palpation/observation, whereas we used observation alone (which probably identifies fewer cows with tail damage; AJ-Gh, personal observation).

It is unclear from this analysis what is driving the high prevalence of tail damage on dairy farms. Neither herd size nor system affected prevalence at the univariable level. Further research is required to establish the risk factors for tail damage on Iranian dairy farms. This should be combined with a standardisation of the tail scoring process and a test of its repeatability and reliability.

#### 4.3.2. Locomotion Scoring

The median herd-level prevalence of lame cows (score ≥ 2) was 33%, lower than the 52.2% reported by Mohamadnia et al. [40] across three Iranian dairy herds but higher than the median of 22% identified by a systematic review of worldwide lameness prevalence [50]. In contrast, the median herd-level prevalence (3.1%) of severely lame cows (LS = 3) was lower than the median prevalence of severe lameness (6.5%) reported in that review. It is clear that there is a significant effort to improve lameness and control it on Iranian dairy farms.

#### 4.3.3. Hock Lesions

As in previous studies [32], we found that swelling and hair loss were much more common than ulceration. The prevalence of hock lesions in this study is similar to those reported on Chinese farms [51] and on farms in British Columbia [20]. Nevertheless, these results indicate that hock lesions are a significant welfare problem on many Iranian farms. We identified a much higher prevalence of hock lesions on freestall farms than on bedded pack farms, consistent with previous studies [52,53], so focusing on freestall farms and identifying why some freestall farms have a much higher level of hock lesions than others could appreciably improve welfare on Iranian freestall farms.

#### 4.3.4. Injuries to Knees, Back, Neck, and Other Body Parts

While injuries can be important indicators of dairy cattle welfare on farms, there is a clear lack of data in the literature regarding the prevalence of such injuries. Our results for knee, back, and neck injuries are consistent with prevalences reported in other systems with intensively managed dairy cows [15,16,54].

#### 4.3.5. Ear Damage

Our study showed that a high number of cows (median of 13.5%) in this survey had damaged ears, and that all farms had cows with damaged ears (range 1.4 to 100%). We speculate that this may have been related to ear tags being pulled out, but ear tags are commonly used worldwide [55], and if they routinely resulted in ear damage in almost 1/8 of tagged cows, we would have anticipated that there would have been previous reports of such damage. Further investigation is required.

#### 4.3.6. Nasal and Ocular Discharges

The median prevalence of cows with severe nasal discharge (6.2%) was consistent with previous reports, which have ranged between 0 and 16.5% [56,57], but the prevalence of ocular discharge (12.8%) is higher than previous reports (0–5% [21,56,57]. This may be due to the environment that predominated on Iranian dairy farms, but this needs confirming by further investigation.

#### 4.3.7. Teat Hyperkeratosis

Mein et al. [36] set targets of ≤20% of animals with both moderate and severe teat hyperkeratosis and <10% of animals having severe teat hyperkeratosis. In our study population, 18/49 farms failed to meet the target for moderate/severe lesions, while 4/49 had too many severe cases. These data suggest that attention to teat health is required on a substantial percentage of Iranian dairy farms.

### 4.4. Human-Animal Relationship

#### 4.4.1. Stockperson Behaviour (Direct Measurement of Human-Animal Relationship)

On most farms (62%), cows were moved from their pens to the milking parlour using a combination of loud noises and hitting, with only 13% of farms using neither approach. This is a higher percentage of issues than reported by Sapkota et al. [58] on New Zealand dairy farms and is more reminiscent of the behaviour recorded by Leon et al. [59] in a slaughterhouse, where physical force (hitting/prodding; 49%) and shouting (13%) were the most common ways to interact with the cows. This high level of aversive stockpersonship during milking (when cows are voluntarily moving) suggests that there may be even more issues at times when cows are more reluctant to move (e.g., entering the trimming chute during hoof trimming; [60]). We need further data on the quality of stockpersonship on Iranian dairy farms and the factors driving it, but it is clear from our interactions with farm staff during the study that lack of training is likely to play a major role.

#### 4.4.2. Avoidance Test (Indirect Measurement of Human-Animal Relationship)

In contrast to our direct assessment of the human-animal relationship, the avoidance test showed that almost 2/3 of cows did not have large flight zones. This is consistent with Waiblinger et al. [61], who found that stockperson behaviour when moving the cows from their pens to the milking parlour was not related to the cows’ avoidance distance. In addition, the high human-to-animal ratio on many of the study farms may also be related to the low avoidance distance [62]. Further investigation is needed to confirm this hypothesis.

## 5. Conclusions

This study assessed different aspects of dairy cattle welfare in 62 Iranian dairy farms in arid and semi-arid regions in Iran. The low prevalence of cows with very low or very high BCS confirms the focus on nutrition management on Iranian dairy farms. Other aspects of welfare, such as body hygiene and skin injuries, were relatively close to what is seen on North American farms. Lameness prevalence and the incidence of hock lesions are also similar to many zero-grazed farms outside Iran but are too high and need more attention. Finally, the extremely high median prevalence of tail damage (60%) indicates a significant welfare issue that needs to be addressed with urgency. Overall, our results indicate that Iranian dairy farmers would hugely benefit from more education regarding dairy cattle welfare.

## Figures and Tables

**Table 1:** The number of animals scored and the number of farms assessed for each welfare measure in a cross-sectional study of 62 Iranian dairy farms.

Domain		Indicator *	No. Animals	No. Farms
Nutrition		BCS ** ^1^	4788	61
Physical Environment	Body Hygiene	Lower Leg ^1^	4803	62
		Upper Leg ^1^	4803	62
		Udder ^1^	4802	62
		Tail ^1^	4803	62
		Hock Hair Loss ^1^	3473	55
	Hock Lesions	Hock Ulcer ^1^	3474	55
		Hock Swelling ^1^	3477	55
Health		Hock ^1^	4804	62
		Knee ^1^	4806	62
	Skin Injuries	Neck ^1^	4806	62
		Back ^1^	4805	62
		Other Parts ^1^	4803	62
		Ear Damage ^1^	2854	47
		Nasal Discharge ^2^	4236	50
		Ocular Discharge ^2^	4346	50
		Teat Hyperkeratosis ^3^	3676	49
		Tail Damage ^1^	4796	61
		Locomotion Score ^4^	14,172	61
Behavioural Interactions		Avoidance Test ^2^	4404	50

* Numbers represent the scoring location: (1) inside pen; (2) feed alley; (3) inside parlour; (4) parlour exit. ** Body Condition Score using the 1 to 5 UK system.

**Table 2:** Distribution of the farm-level prevalence of animals with different welfare problems in a cross-sectional study of 62 Iranian dairy farms.

Welfare Domain		Measure	Mean	Lower CI	Upper CI	First Quartile	Median	Third Quartile	Minimum	Maximum	No. of Farms
Nutrition (BCS)		Lean Cows	0.49	0.19	0.79	0	0	0.91	0	7.84	61
		Fat Cows	0.21	0.00	0.43	0	0	0	0	5.13	61
Health	Tail Damage		59.1	53.7	64.5	43.1	60.0	76.9	6.7	100	61
	Locomotion Score 2 (Clinically Lame)		31.3	28.1	34.5	22.5	30.8	37.1	7.4	66.8	61
	Locomotion Score 3 (Severely Lame)		4.04	30.3	5.04	1.20	3.0	6.50	0	22.1	61
	Hock (dorsal joint) Lesions	Hair loss	38.3	31.7	44.9	17.8	36.0	51.8	0	97.9	55
		Swelling	41.5	35.4	47.6	23.5	40.7	60.9	0	87.9	55
		Ulcer	4.25	2.43	6.07	0	1.92	6.63	0	41.0	55
	Skin Injuries	Knee (carpal joint)	28.0	21.2	34.9	6.33	16.8	46.7	0	97.4	62
		Neck	7.91	5.17	10.7	0	3.50	9.84	0	38.3	62
		Back	5.64	4.24	7.05	2.20	4.30	7.02	0	31.1	62
		Other parts	25.3	21.3	29.4	13.7	23.4	32.3	0	81.8	62
		Ear Damage	20.5	14.7	26.3	7.25	13.5	25.0	1.41	100	45
	Discharges	Nasal	8.89	5.84	11.9	1.70	6.20	11.6	0	65.2	58
		Ocular	14.5	11.6	17.5	6.50	12.8	19.7	0	52.4	60
	Teat Hyperkeratosis		23.0	15.9	30.1	4.15	8.73	42.3	0	91.2	48
Environment	Dirty Body Parts	Lower Leg	96.8	94.4	99.1	98.9	100	100	48.6	100	62
		Upper Leg	86.8	81.8	91.7	80.0	95.7	100	21.1	100	62
		Udder	74.3	67.4	81.2	59.0	83.6	97.8	7.62	100	62
		Tail	86.0	80.9	91.1	81.7	95.1	98.9	24.8	100	62
Behavioural Interaction		Avoidance 0	39.2	35.1	43.4	28.0	38.9	53.0	4.30	79.2	60
		Avoidance 1	24.7	21.6	27.8	15.2	22.9	33.7	4.20	52.7	60
		Avoidance 2	36.1	31.7	40.5	21.1	36.4	46.6	3.70	70.4	60

**Table 3:** The effect of different farming systems and farm sizes on the prevalence of tail damage, lameness, and hock lesions in a cross-sectional study of 62 Iranian dairy farms.

Farming System					
Indicators	Farming System ^a^	Exp (Coefficient)	95% CI Exp (Coefficient)		*p*-Value
			Lower	Upper	
Tail Damage	Freestall	1.03	0.77	1.36	0.86
	Bedded-Pack ^b^	.	.	.	-
Lameness	Freestall	0.91	0.74	1.12	0.37
	Bedded-Pack ^b^	.	.	.	-
Hock Hair loss	Freestall	3.82	2.37	6.13	<0.001
	Bedded-Pack ^b^	.	.	.	.
Hock Swelling	Freestall	2.56	1.60	4.10	<0.001
	Bedded-Pack ^b^	.	.	.	.
Hock Ulcer	Freestall	2.35	1.18	4.70	0.02
	Bedded-Pack ^b^	.	.	.	.
**Farm Size**					
Tail Damage	Small	1.32	0.71	2.46	0.37
	Medium	0.97	0.66	1.45	0.89
	Large ^b^	.	.	.	.
Lameness	Small	0.83	0.53	1.28	0.39
	Medium	0.87	0.63	1.21	0.40
	Large ^b^	.	.	.	.
Hock Hair loss	Small	0.63	0.28	1.45	0.27
	Medium	0.55	0.24	1.29	0.16
	Large ^b^	.	.	.	.
Hock Swelling	Small	0.76	0.36	1.59	0.45
	Medium	0.83	0.39	1.78	0.63
	Large ^b^	.	.	.	.
Hock Ulcer	Small	0.96	0.37	2.48	0.93
	Medium	0.56	0.21	1.50	0.24
	Large ^b^	.	.	.	.

^a^—farm is considered as a random effect in the model. ^b^—this coefficient is set to zero because it is redundant, as it is the reference category for the odds ratio.

**Table 4:** Distribution of herd-level prevalence of animals with different welfare issues in free-stall and open-shed farms in a cross-sectional study of 62 Iranian dairy farms.

Measure		Category	Mean	Lower 95% CI	Upper 95% CI	First Quartile	Median	Third Quartile	Minimum	Maximum
Tail Damage		Free-stall	55.1	46.9	63.3	37.5	57.3	69.6	16.1	100
		Bedded-pack	60.6	53.3	67.9	46.6	55.5	80.9	6.7	100
Lameness		Free-stall	39.6	32.9	46.3	28.1	37.6	46.7	10.1	85.7
		Bedded-pack	31.4	27.3	35.4	24.1	30.8	38.6	8.2	56.5
Hock (dorsal joint) Lesions	Hair loss	Free-stall	56.2	47.5	64.9	43.7	51.7	74.3	16.7	97.9
		Bedded-pack	25.4	18.7	32.1	14.7	23.3	36.0	0	95.5
	Swelling	Free-stall	54.5	46.1	62.9	38.3	54.6	66.2	22.9	87.9
		Bedded-pack	32.6	25.5	39.7	17.5	28.8	45.3	0	73.3
	Ulcer	Free-stall	7.09	3.18	11.0	1.10	3.75	10.3	0	41.0
		Bedded-pack	2.35	1.12	3.58	0	1.18	3.51	0	17.1

## Data Availability

Data can be available based upon request from the corresponding author.

## Supplementary Materials

The following supporting information can be downloaded at: https://www.mdpi.com/article/10.3390/ani15030359/s1.
