# Supplementary material for: An Investigation of Dairy Cattle Welfare in Commercial Iranian Farms: Results from Animal- and Stockperson-Based Measures
Source: Animals (Basel). 2025 Jan 27;15(3):359. doi: 10.3390/ani15030359 (PMC11815728; doi:10.3390/ani15030359)
Supplement: Supplementary file 1 [file animals-15-00359-s001.zip › animals-3349053-supplementary.pdf]

Farm Name: Farm Owner: Date of Visit:

Observer: Time of Audit: Temperature:

Form Z

Environment/ Management

Water Trough Length	Max. waiting time for parlor
Water Trough Hygiene	Parlor ventilation
Feed Trough Length	Parlor floor
Feed Trough Hygiene	Noise level in parlor
Shelter above the trough	Track condition
Choice of water Temp.	Distance to water after parlor
Dam-calf relation	Safe/clean bedding
Comfortable calving pens	Barn ventilation
Hoof trimming frequency	Animal density
Foot bath frequency	Manure removal
Personnel behavior	Shelter above the resting area

Free-stall design

Stalls with manure contamination	%
Dirt alley	Y/N
Stocking density	%
Brisket locator	Y/N
Width	Cm
Neck rail height	Cm
Neck rail distance to the rear curb	Cm
Adjustable neck rail	Y/N
Position of animals in the cubicles	

Farm Records

Total number of mastitis cases	
Total number of lameness cases	
Total mortality and culling	
Average Days Open	
Average Milk Yield (3.5% CMF)	
Average Fat (annual)	
Average Milking Days (DIM)	
Milk SCC	
Average Culling in the lactating herd	
Average Calf Mortality (<3 months)	
Calf mortality at birth	

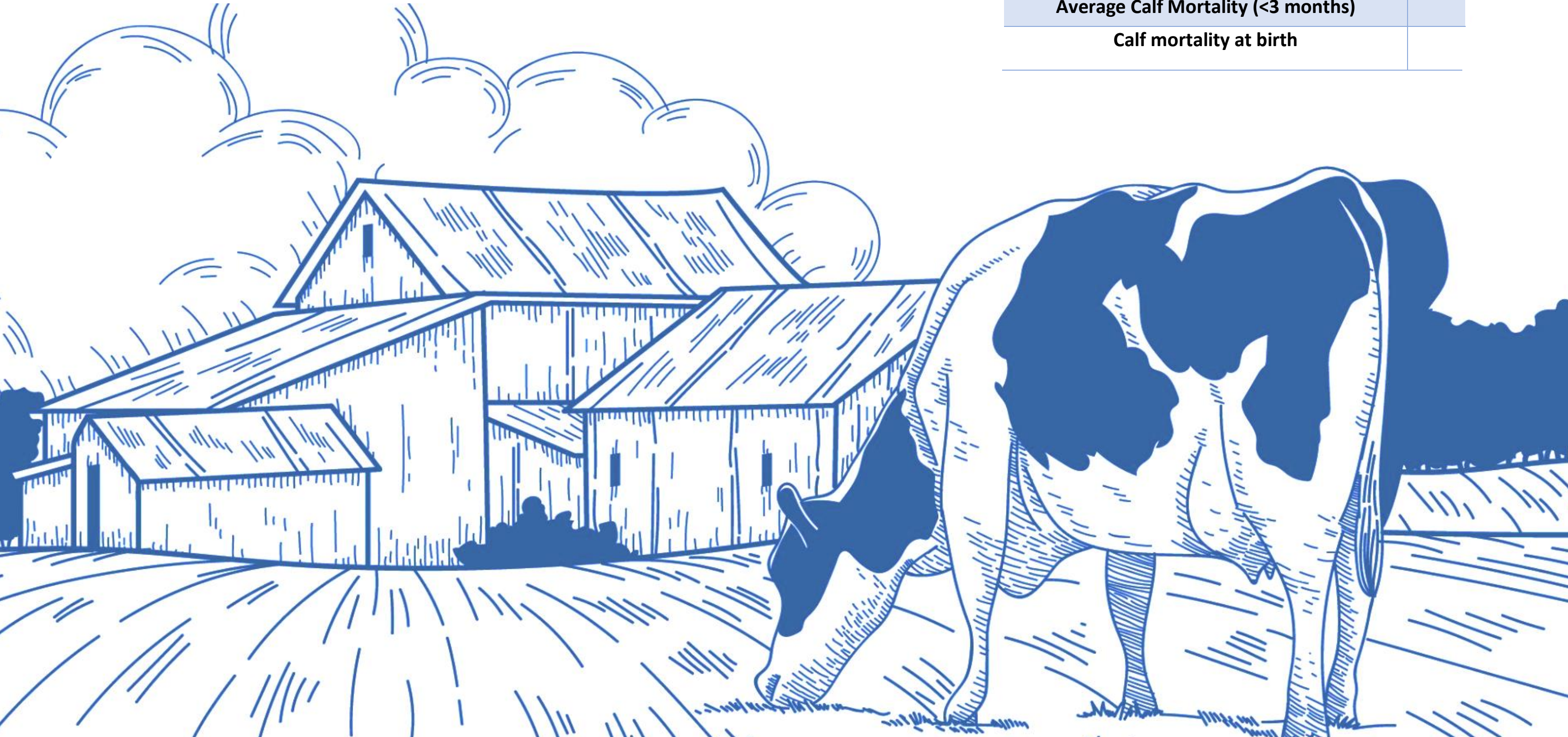

Form A

Cow No.	Hygiene				Injuries					BCS
	Lower Leg	Upper Leg	Tail	Udder	Hock	Knee	Neck	back	Other parts	

Cow No.	Hygiene				Injuries					BCS
	Lower Leg	Upper Leg	Tail	Udder	Hock	Knee	Neck	back	Other parts	

Form B

Cow No.	Avoidance	Discharge	
		Nasal	Ocular

Cow No.	Avoidance	Discharge	
		Nasal	Ocular

Form C

Cow No.	Lameness	NZ BCS	Tail

Cow No.	Lameness	NZ BCS	Tail

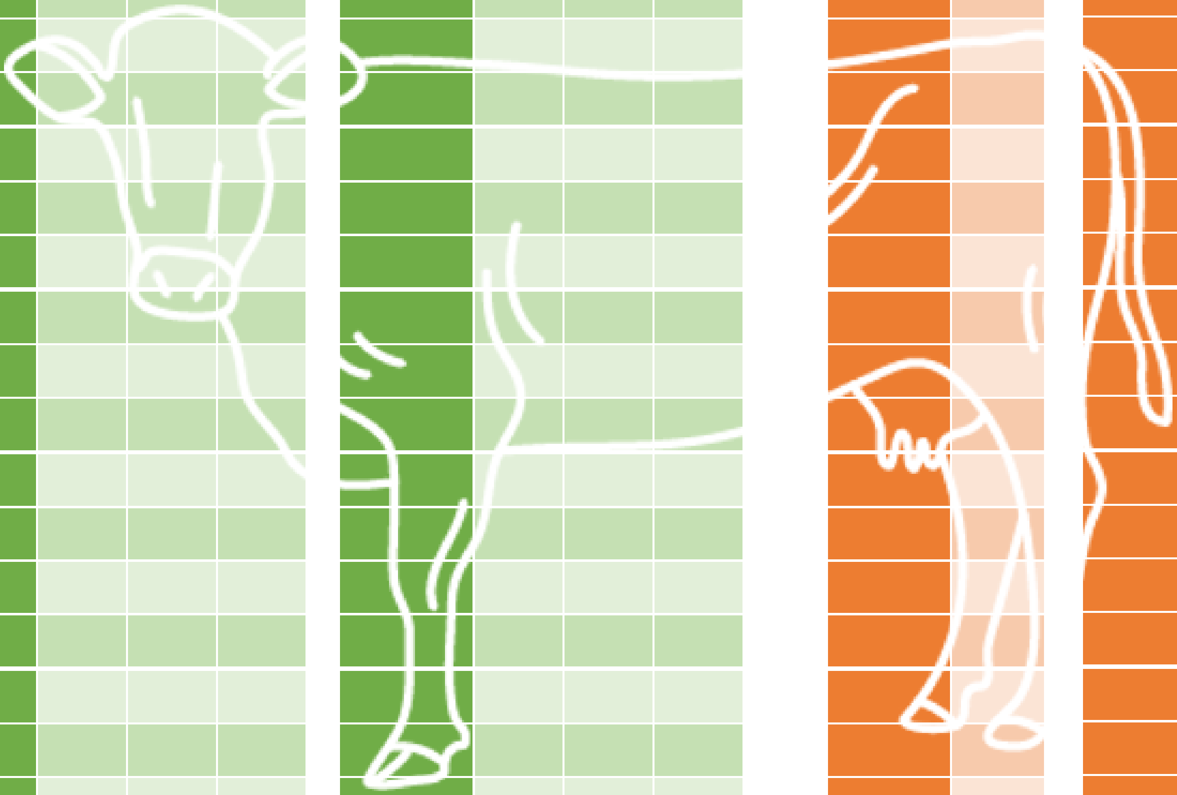

Cow No.	Temperature

Cow No.	Temperature

Papers used to develop a welfare assessment protocol that could be used on intensive dairy cattle farms in Iran

North America:

Cook, N.B., Hess, J.P., Foy, M.R., Bennett, T.B. and Brotzman, R.L., 2016. Management characteristics, lameness, and body injuries of dairy cattle housed in high-performance dairy herds in Wisconsin. *Journal of Dairy Science*, *99*(7), pp.5879-5891.

Denis-Robichaud, J., Kelton, D., Fauteux, V., Villettaz-Robichaud, M. and Dubuc, J., 2020. Accuracy of estimation of lameness, injury, and cleanliness prevalence by dairy farmers and veterinarians. *Journal of dairy science*, *103*(11), pp.10696-10702.

Nash, C.G.R., Kelton, D.F., DeVries, T.J., Vasseur, E., Coe, J., Heyerhoff, J.Z., Bouffard, V., Pellerin, D., Rushen, J., De Passillé, A.M. and Haley, D.B., 2016. Prevalence of and risk factors for hock and knee injuries on dairy cows in tiestall housing in Canada. *Journal of dairy science*, *99*(8), pp.6494-6506.

Solano L., H.W. Barkema, E.A. Pajor, S. Mason, S.J. LeBlanc, J.C. Zaffino Heyerhoff, C.G.R. Nash, D.B. Haley, E. Vasseur, D. Pellerin, J. Rushen, A.M. de Passillé, K. Orsel, 2015. Prevalence of lameness and associated risk factors in Canadian Holstein-Friesian cows housed in freestall barns, Journal of Dairy Science, 98 (10), https://doi.org/10.3168/jds.2015-9652.

Robichaud, M.V., Rushen, J., De Passillé, A.M., Vasseur, E., Orsel, K. and Pellerin, D., 2019. Associations between on-farm animal welfare indicators and productivity and profitability on Canadian dairies: I. On freestall farms. *Journal of Dairy Science*, *102*(5), pp.4341-4351.

von Keyserlingk, M.A., Barrientos, A., Ito, K., Galo, E. and Weary, D.M., 2012. Benchmarking cow comfort on North American freestall dairies: Lameness, leg injuries, lying time, facility design, and management for high-producing Holstein dairy cows. *Journal of dairy science*, *95*(12), pp.7399-7408.

Winckler, C., Tucker, C.B. and Weary, D.M., 2015. Effects of under-and overstocking freestalls on dairy cattle behaviour. *Applied Animal Behaviour Science*, *170*, pp.14-19.

Europe:

Potterton S. L. Green M. J. Harris J. Millar K. M. Whay H. R. Huxley J. N. (2011b) Risk factors associated with hair loss, ulceration, and swelling at the hock in freestall-housed UK dairy herds. *Journal of Dairy Science* 94, 2952 –2963

Whay, H.R., Main, D.C.J., Green, L.E. and Webster, A.J.F., 2003. Assessment of the welfare of dairy caftle using animal-based measurements: direct observations and investigation of farm records. *Veterinary record*, *153*(7), pp.197-202.

Zuliani, A., Mair, M., Kraševec, M., Lora, I., Brscic, M., Cozzi, G., Leeb, C., Zupan, M., Winckler, C. and Bovolenta, S., 2018. A survey of selected animal-based measures of dairy cattle welfare in

the Eastern Alps: Toward context-based thresholds. *Journal of dairy science*, *101*(2), pp.1428-1436.

New Zealand:

Laven, R.A. and Fabian, J., 2016. Applying animal-based welfare assessments on New Zealand dairy farms: Feasibility and a comparison with United Kingdom data. *New Zealand Veterinary Journal*, *64*(4), pp.212-217.

Sapkota, S., Laven, R., Müller, K. and Kells, N., 2020. Animal welfare assessment: can we develop a practical, time-limited assessment protocol for pasture-based dairy cows in New Zealand?. *Animals*, *10*(10), p.1918.

Webster, J.R., Schütz, K.E., Sutherland, M.A., Stewart, M. and Mellor, D.J., 2015. Different animal welfare orientations towards some key research areas of current relevance to pastoral dairy farming in New Zealand. *New Zealand Veterinary Journal*, *63*(1), pp.31-36.
